# In Vitro Growth- and Encystation-Inhibitory Efficacies of Matcha Green Tea and Epigallocatechin Gallate Against *Acanthameoba Castellanii*

**DOI:** 10.3390/pathogens9090763

**Published:** 2020-09-17

**Authors:** Ameliya Dickson, Elise Cooper, Lenu B. Fakae, Bo Wang, Ka Lung Andrew Chan, Hany M. Elsheikha

**Affiliations:** 1 School of Veterinary Medicine and Science, Faculty of Medicine and Health Sciences, University of Nottingham, Loughborough LE12 5RD, UK; svyad3@exmail.nottingham.ac.uk (A.D.); svyec7@exmail.nottingham.ac.uk (E.C.); stxlbf@exmail.nottingham.ac.uk (L.B.F.); 2 School of Mathematics and Actuarial Science, University of Leicester, Leicester LE1 7RH, UK; bo.wang@leicester.ac.uk; 3 Institute of Pharmaceutical Science, King’s College London, London SE1 9NH, UK; ka_lung.chan@kcl.ac.uk

**Keywords:** *Acanthameoba castellanii*, *Camellia sinensis*, matcha green tea, epigallocatechin gallate, trophozoite, cyst

## Abstract

We examined the inhibitory effect of matcha green tea (*Camellia sinensis*) and epigallocatechin gallate (EGCg; the most abundant catechin in tea) on the vegetative growth and encystation of *Acanthamoeba castellanii* T4 genotype. The sulforhodamine B (SRB) stain-based colorimetric assay and hemocytometer counting were used to determine the reduction in *A. castellanii* trophozoite proliferation and encystation, in response to treatment with *C. sinensis* or EGCg. Fourier transform infrared (FTIR) microscopy was used to analyze chemical changes in the trophozoites and cysts due to *C. sinensis* treatment. Hot brewed and cold brewed matcha inhibited the growth of trophozoites by >40% at a 100 % concentration. EGCg at concentrations of 50 to 500 µM significantly inhibited the trophozoite growth compared to control. Hot brewed matcha (100% concentration) also showed an 87% reduction in the rate of encystation compared to untreated control. Although 500 µM of EGCg increased the rate of encystation by 36.3%, 1000 µM reduced it by 27.7%. Both percentages were not significant compared to control. *C. sinensis* induced more cytotoxicity to Madin Darby canine kidney cells compared to EGCg. FTIR chemical fingerprinting analysis showed that treatment with brewed matcha significantly increased the levels of glycogen and carbohydrate in trophozoites and cysts.

## 1. Introduction

Due to the relative rarity of clinical *Acanthamoeba castellanii* infections, there are currently limited treatment options available. There is a critical need for new drugs that are potent and safe to use in *Acanthamoeba* keratitis treatment. Several studies have reported cytotoxicity to human fibroblasts [1] and corneal keratocytes [2] by a biguanide antiseptic agent. These drugs also require prolonged treatment, on average lasting up to six months [3]. Treatment is further complicated by the presence of amoebic cysts, which prove to be very resistant to many of the currently available drugs. This lack of optimal treatment has inspired researchers to investigate the use of naturally occurring compounds as an alternative to current medicines, or as a reciprocal treatment to help alleviate or prevent *A. castellanii* infections. Among a plethora of natural compounds tested, a few examples include, *Ipomoea* spp., *Kaempferia galanga*, *Cananga odorata*, oakmoss (a natural fragrance ingredient), hexane fraction of *Pterocaulon polystachyum*, ethyl acetate extract of Limouni olive leaf, resveratrol and curcuminoids were found to be amoebicidal [4].

*Camellia sinensis* leaves are cultivated to produce traditional green tea and matcha, which have been shown to inhibit the growth of various pathogens [5], including *A. castellanii* [6]. These antimicrobial effects have been linked to catechins [7], which have been shown to have anti-protozoal activities. For example, catechins can inhibit arginine kinase, which is a key metabolic enzyme in *Trypanosoma cruzi*. The catechins, gallocatechin gallate (GCG, at 0.12 pM) and epigallocatechin gallate (EGCg at 0.53 pM) were found to lyse more than 50% of the trypomastigotes [8]. In another study, EGCg inhibited the growth of bovine *Babesia* spp. and caused degeneration of the parasites at IC_50_ of 18 µm (*Babesia bovis*) and 25 µm (*Babesia bigemina*) [9]. Matcha green tea has a particularly high concentration of catechins, including EGCg, compared with traditional green tea, due to the unique way the tea plant leaves are cultivated and processed [10]. Given these differences and the fact that matcha tea powder has more physiological benefits than green tea, it seems plausible to investigate the anti-acanthamoebic effects of the matcha tea powder.

Therefore, in this study, we sought to examine the inhibitory effects of *C. sinensis* (matcha) and EGCg on the growth and encystation of *A. castellanii* T4 strain. Overall, our data showed that *C. sinensis* significantly inhibited trophozoite and cystic stages of *A. castellanii*, which was accompanied with significant changes in the chemical composition of the amoeba. EGCg had an inhibitory effect on trophozoite growth only.

## 2. Results

### 2.1. Level of Growth Inhibition of A. castellanii Trophozoites

**Figure 1:**
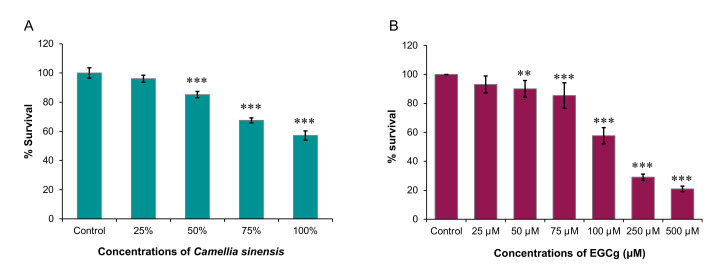
Inhibitory effect of hot brewed matcha and EGCg on the growth of *A. castellanii* trophozoites. (**A**) *A. castellanii* culture was incubated with different concentrations of hot brewed matcha for three days. The growth rate was assessed with sulforhodamine B (SRB) assay. Data (percent survival) are the percentages of SRB absorbance reduction relative to that of the untreated control. Matcha exerted a significant reduction in the trophozoite’s growth at 50% (∗∗∗ *p* = 1.2 × 10^−5^), 75% (∗∗∗ *p* = 2.2 × 10^−16^) and 100% (∗∗∗ *p* < 2 × 10^−16^), compared to untreated control group. (**B**) *A. castellanii* culture was incubated with the indicated concentrations of epigallocatechin gallate (EGCg) for 3 days. The growth rate was assessed with manual counting using a hemocytometer. Data represent the percentages of survival of treated trophozoites compared to control trophozoites. EGCg had a significant inhibitory effect on the growth of *A. castellanii* trophozoites, at 50 µM (∗∗ *p* = 0.0017), 75 µM (∗∗∗ *p* = 1.6 × 10^−8^), 100 µM (∗∗∗ *p* < 2 × 10^−16^), 250 µM (∗∗∗ *p* < 2 × 10^−16^), and 500 µM (∗∗∗ *p* < 2 × 10^−16^) compared to untreated control group.

The survival rate of *A. castellanii* treated with hot brewed matcha showed a dose-dependent inhibitory effect on the growth of trophozoites (Figure 1A). A Dunnett’s test revealed a significant effect of matcha on the rate of trophozoite growth, at 50% (*p* = 1.2 × 10^−5^), 75% (*p* = 2.2 × 10^−16^) and 100% (*p* < 2 × 10^−16^), compared to untreated control trophozoites. At a concentration of 100%, matcha caused a ~42% reduction in the growth of trophozoites. However, there was no significant effect at the lowest tested concentration (25%) of matcha (*p* = 0.5087). Cold brew matcha produced very similar results to those obtained by testing the effect of hot brew matcha.

In comparison to negative control, manual counting showed a clear reduction in the proliferation of *A. castellanii* trophozoites in response to treatment with EGCg in a dose-dependent manner (Figure 1B). The 500 µM concentration of EGCg appears to be the most potent (*p* < 2 × 10^−16^). Moreover, significant inhibitory effects of EGCg on the rate of trophozoite growth were observed at 50 µM (*p* = 0.0017), 75 µM (*p* = 1.6 × 10^−8^), 100 µM (*p* < 2 × 10^−16^), and 250 µM (*p* < 2 × 10^−16^). However, *A. castellanii* trophozoites showed less susceptibility at the lowest EGCg concentration, 25 µM, where result was not significantly different from that of the negative control (*p* = 0.1646). We also investigated the anti-amoebic properties of EGCg using the sulforhodamine B (SRB) assay. However, results showed a positive correlation between colorimetric absorbance and EGCg concentrations. All EGCg-treated wells had higher absorbance values compared to the negative control. Considering the interference of the EGCg with the colorimetric measurement, we decided not to include results obtained by SRB analysis in this study. 

### 2.2. Inhibitory Effect on A. castellanii Encystation

**Figure 2:**
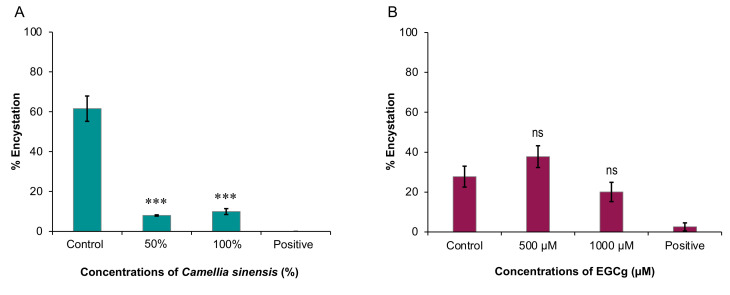
The anti-encystation activity of hot brewed matcha and EGCg. (**A**) A significant inhibitory effect of hot brewed matcha on the rate of encystation was detected at concentrations 50% (∗∗∗ *p* = 4.1 × 10^−6^) and 100% (∗∗∗ *p* = 5.2 × 10^−6^). (**B**) The results obtained by EGCg at 500 µM and 1000 µM concentrations were not significantly (ns) different from the control (*p* = 0.0567 and 0.1522, respectively).

As shown in Figure 2A, a significant inhibitory effect on the rate of encystation was detected at concentrations 50% (*p* = 4.1 × 10^−6^) and 100% (*p* = 5.2 × 10^−6^) of hot brewed matcha. These results show that matcha has anti-encystation activity. As expected, the positive control (PMSF) prevented any cysts from forming. Although EGCg at 1000 µM concentration performed better than the 500 µM concentration, results obtained by both concentrations were not significantly different from the control (*p* = 0.1522 and 0.0567, respectively). Surprisingly, EGCg at 500 µM concentration seemed to induce a marginally significant (*p* = 0.0567) increase in encystation rate compared to the control (Figure 2B). Again, PMSF caused significant inhibition in encystation.

### 2.3. Cytotoxicity of C. sinensis and EGCg on Mammalian Cells

**Figure 3:**
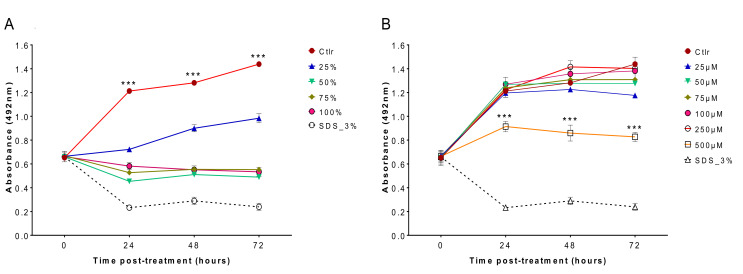
Cytotoxic activity of hot brewed matcha and EGCg on Madin Darby canine kidney (MDCK) cells using the SRB assay. (**A**) The average growth rates at the indicated concentrations of matcha. The results show that matcha was cytotoxic at all the tested concentrations (*** *p* < 0.001). (**B**) The average growth rates at the indicated concentrations of EGCg. The results suggest that EGCg has a cytotoxic effect at 500 µM concentration starting at 24 h post treatment to the end of the experiment (*** *p* < 0.001), and that no cytotoxic effects were detected at lower concentrations.

The cytotoxicity of matcha and EGCg against MDCK cells was investigated. The rate of cell survival following treatment with 25%, 50%, 75% and 100% of matcha at 24, 48 and 72 h was significantly lower than that of the control, untreated cells (*p* < 0.001), indicating that *C. sinensis* was cytotoxic to MDCK cells at the tested concentrations (Figure 3A). The survival rate of MDCK cells exposed to 25% matcha was higher than among cells exposed to the higher concentrations (50%, 75% and 100%; *p* < 0.001). The rate of MDCK cell survival following treatment with EGCg at 500 μM was significantly lower than that treated with other concentrations and the untreated control cells at 24, 48 and 72 h (Figure 3B). These results suggest that EGCg has a clear cytotoxic effect at 500 µM concentration starting at 24 h post treatment, however no significant cytotoxicity was observed at lower EGCg concentrations.

### 2.4. Chemical Differences between Treated and Untreated Trophozoites

**Figure 4:**
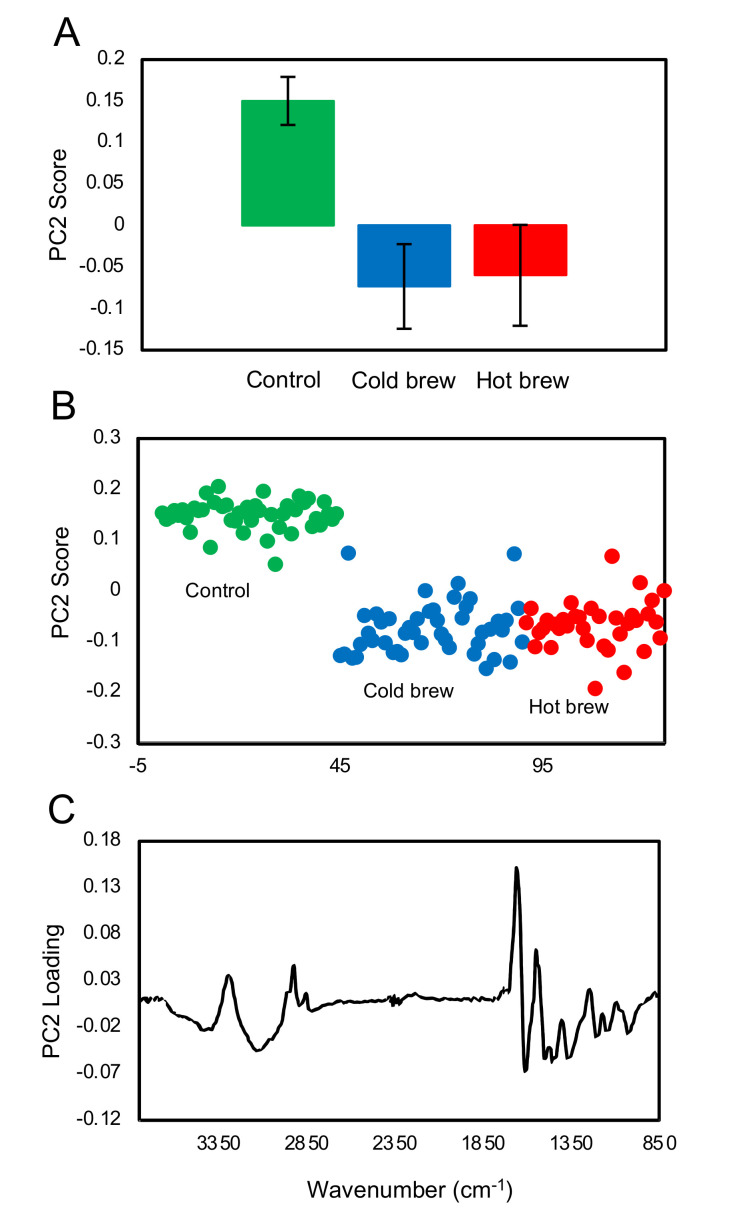
PCA of the spectra collected from individual untreated (control) and treated (cold brewed and hot brewed matcha) trophozoites. (**A**) The average value of the PC2 scores of the control samples versus samples treated with cold brewed and hot brewed matcha. The error bar denotes the +/− standard deviation of the data set. (**B**) The PC2 scores of all spectra with green, blue and red dots representing untreated trophozoites and trophozoites treated with cold brewed and hot brewed matcha, respectively. (**C**) The loading plot of PC2 highlighting the features that distinguished the treated and untreated groups.

The potency exhibited by matcha brew against trophozoite and cyst stages has prompted us to investigate the effect of hot brewed matcha on the chemical composition of trophozoites and cysts using FTIR microscope. Spectra of control, cold brew- and hot brew-treated trophozoites were analyzed using principal component analysis (PCA). First, we compared the untreated to treated trophozoites. PC1 (not shown) did not produce separation between the three group of trophozoite samples, because there were several trophozoites, particularly within the treated group, which have shown large differences in the spectral profile as a result of strong light scattering. These large differences were associated with the morphological changes of trophozoites in response to the exposure to matcha. The second most significant principal component (PC2), shown in Figure 4A,B, shows that the control (untreated) and treated trophozoites were well separated with statistical significance of *p* < 0.0001. The loading plot (Figure 4C) of PC2 shows that treated trophozoites exhibited a number of specific changes in the protein region 1700–1500 cm^−1^ which is sensitive to protein conformation, negative peaks at 1450 cm^−1^ and 1370 cm^−1^ (δ (CH_3_) and ν (COO^−^)), 1205 cm^−1^, 1150 cm^−1^ and 1023 cm^−1^ (carbohydrate region) signifying complex chemical changes within the trophozoites due to treatment.

### 2.5. Spectral Comparison of Trophozoites Treated with Hot vs. Cold Brewed Matcha

**Figure 5:**
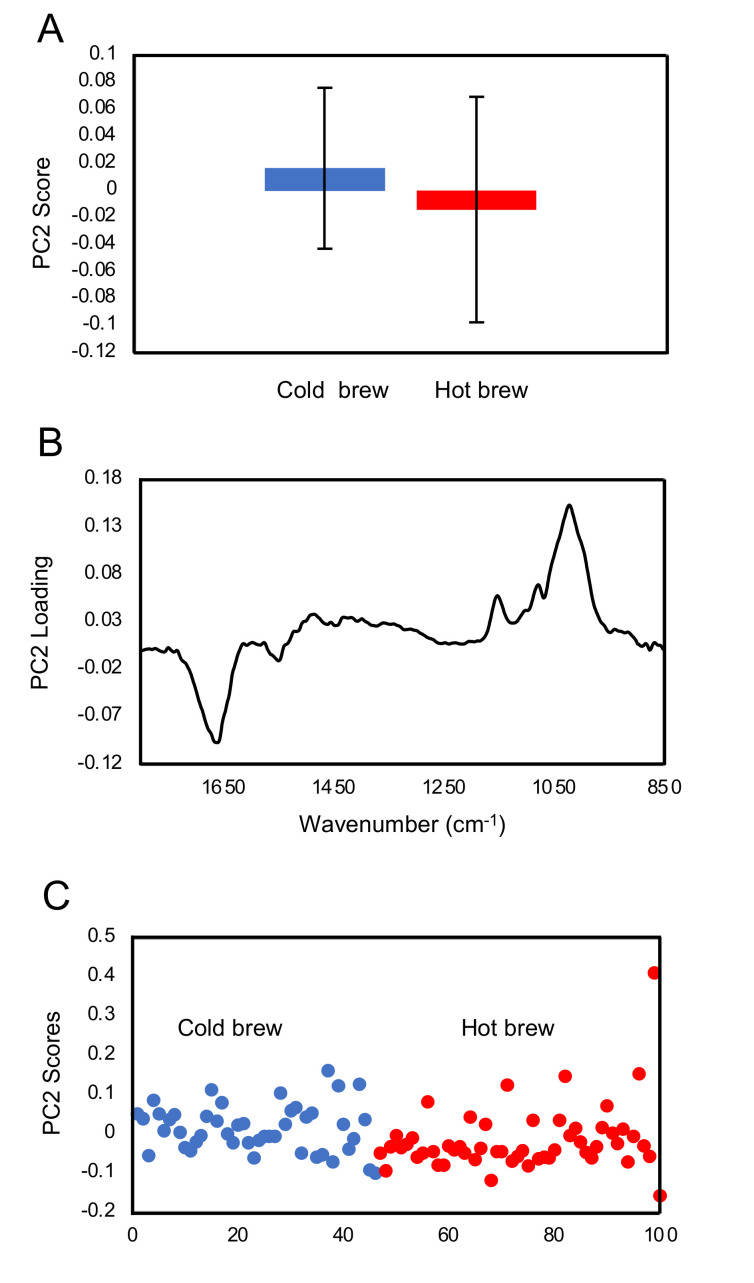
PCA of spectra collected from single trophozoites treated with cold or hot brewed matcha. (**A**) The average value of the PC2 scores of trophozoites treated with cold- and hot-brewed matcha, denoted by blue and red colors, respectively. The error bar represents +/− standard deviation of the data set. (**B**) The loading plot of PC2 highlighting the features that distinguished the cold brew and hot brew matcha-treated groups. (**C**) The PC2 scores of all spectra with blue and red dots representing trophozoites treated with cold brewed and hot brewed matcha, respectively.

A comparison between the cold brewed and hot brewed matcha treatments was carried out. PC1 (not shown) produced some separations between the two type of treatments, however, the loading spectrum has shown the same feature, presumably due to light scattering. This suggests that there were slight morphological differences between the trophozoites treated with cold brewed or hot brewed matcha. As shown in Figure 5A, the second most important principal component (PC2) shows that the difference between the cold brewed and hot brewed matcha treatment was small but significant with a *p* value of <0.05 when the analysis was focused on the fingerprint region (1800–850 cm^−1^). However, when the analysis was performed over the full mid-IR range, there was no significant difference between the two treatments. This indicates that the small differences between cold and hot brews are found in the fingerprint region. The loading plot of PC2 (Figure 5B) shows that the differences was mainly found in the carbohydrate region between 1150–900 cm^−1^. The three prominent peaks at 1150, 1080 and 1023 cm^−1^ coincided with the peaks of glycogen. Trophozoites containing high glycogen contents are shown with a high PC2 score in Figure 5C.

### 2.6. Effect of Matcha on the Chemical Composition of A. castellanii Cysts

**Figure 6:**
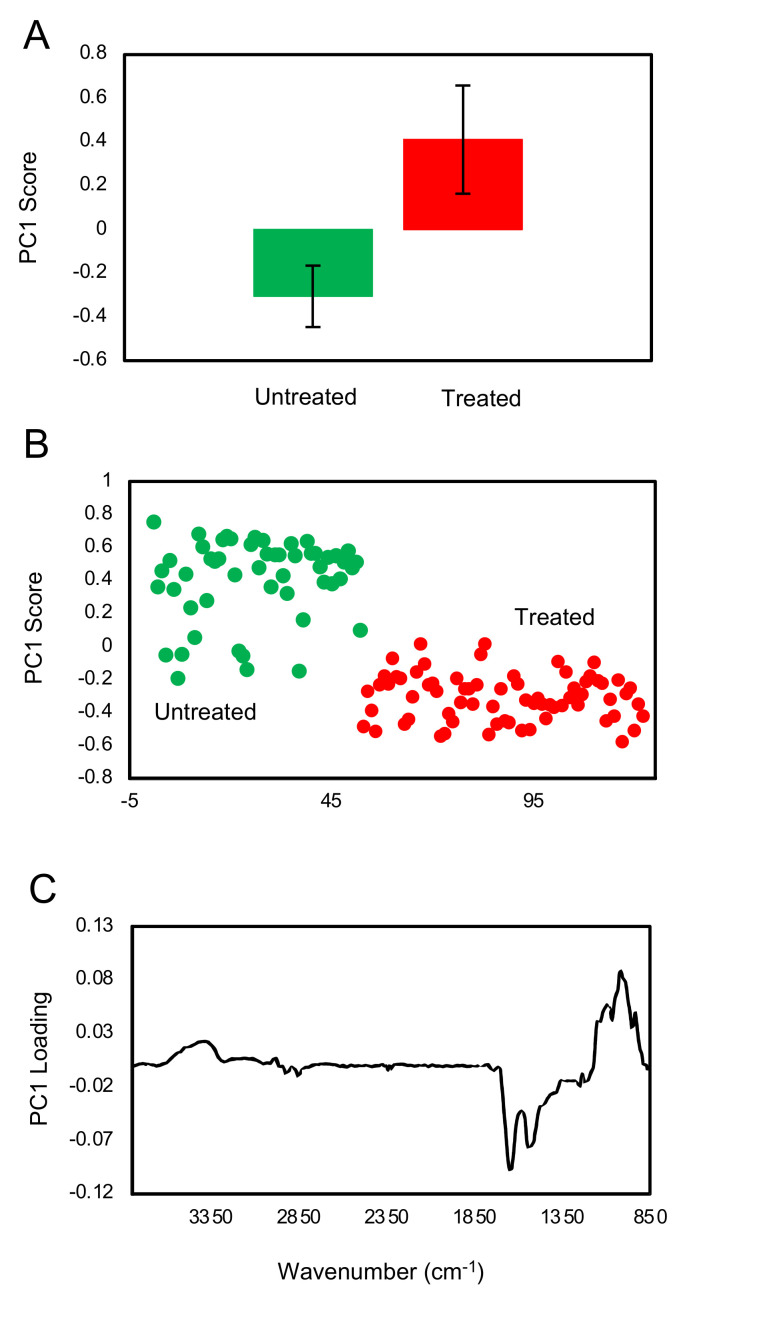
PCA of FTIR spectra collected from individual untreated and hot brew matcha-treated cysts. (**A**) The average value of the PC1 scores of the untreated and treated samples, denoted by green and red colors, respectively. The error bar represents the +/− standard deviation of the data set. (**B**) The PC1 scores of all spectra with green and red colors representing the untreated cysts and cysts treated with hot brewed matcha, respectively. (**C**) The loading plot of PC1 highlighting the features that distinguished the two groups.

FTIR spectra of treated and untreated individual *A. castellanii* cysts were collected using FTIR microscope, and the results were analyzed using PCA. As shown in Figure 6A,B, the treated and untreated cysts are well separated by the first principal component (PC1), supported with a high statistical significance of *p* < 0.0001. The loading plot (Figure 6C) of PC1 shows that the treated cysts had higher carbohydrate peaks in 1150–900 cm^−1^ region, relative to the protein amide I and amide II peaks at 1640 and 1535 cm^−1^, respectively.

## 3. Discussion

In this study, we tested the efficacy of *Camellia sinensis* (matcha) and epigallocatechin gallate (EGCg) against trophozoites and cyst stages of *A. castellanii*. Matcha was found to significantly inhibit the growth of *A. castellanii* trophozoites when brewed in cold or hot water at a concentration of 0.01 g matcha powder/mL distilled water compared with non-treated trophozoites. Results of SRB assay recorded from trophozoites treated with a range of concentrations of matcha, and particularly those treated with the highest concentration (100%) had a significantly lower optical density (i.e., reduced growth rate of trophozoites) compared with those that were incubated in PYG medium alone. Previous studies showed that antioxidant capabilities and the content of phenolic compounds of green tea and matcha tea are increased with increasing the water temperature used for the preparation of the infusion/brew, with the highest values achieved at 100 °C [10] and 90 °C [11]. In our study, similar results were obtained by testing the effect of hot and cold brews, suggesting that brewing temperature does not seem to influence the matcha anti-acanthamoebic activity, or that the effector substance(s) in matcha are not affected by heat. Based on these results, we tested the effect of hot brewed matcha on the encystation rate, which was greatly reduced in cultures exposed to encystation solution containing hot brewed matcha as a solvent compared with cultures exposed to standard encystation solution only.

The lower concentration of matcha (50%) showed slightly more inhibition of cyst formation compared to the higher concentration (100%). Although there is no direct explanation of the mechanism causing this, a previous study found that matcha extracts reduced the amount of *Entamoeba histolytica* cysts in mouse feces by the same rate (84%), in both lower (100 mg/kg) and higher (150 mg/kg) concentrations [12]. This may indicate that matcha does not show a dose dependent effect on encystation. Overall, these data show that matcha has an inhibitory activity against both trophozoite and cystic stages of *A. castellanii*. This result is consistent with that reported previously about amoebicidal activity of green tea brew against the trophozoite and cystic forms of *A. castellanii* [6]. The comparable efficacies of brews obtained from matcha and green tea against *A. castellanii* suggest that the different cultivation conditions and processing of tea plant leaves (*C. sinensis*) for the production of green tea and matcha [10] are less likely to influence the anti-acanthamoebic properties of *C. sinensis*.

Our results also showed that EGCg had a significant inhibitory effect against *A. castellanii* trophozoite growth where 500 µM EGCg inhibited *A. castellanii* trophozoite growth by 79.1% (*p* < 2 × 10^−16^). The same inhibitory effect of EGCg was not observed against *A. castellanii* encystation. These results are in agreement with previous studies which reported EGCg potency against the different life cycles stages of various parasites. For example, 50 µM EGCg completely cleared *Babesia bovis* and *B. bigemina* from the blood within 72 h [9], 12 µM EGCg limited the growth of *Leishamania amazonenesis* by 83.6% within 72 h [13], and 200 µg/mL EGCg killed nearly all *Plasmodium berghei* sporozoites within 12 hrs [14]. It might seem unusual to see both matcha and EGCg have inhibitory activity against the proliferation of trophozoites but differ in their effect on the cystic stage. EGCg did not show significant effect against the cystic stage, although matcha which contains EGCg exhibited a clear cysticidal activity. This result suggests that cysticidal activity of matcha must have been attributed to other molecules, rather than EGCg, present in matcha. Therefore, it is likely that catechins or other substances within matcha are responsible for the observed inhibitory effect of brewed matcha on the cyst formation.

The mechanism underpinning the anti-acanthamoebic activity of matcha or EGCg has not been investigated in the present study. However, catechins found in traditional green tea leaves have been shown to have antiprotozoal effects on other organisms, through the inhibition of arginine kinase in *Trypanosoma cruzi* [8], production of intracellular H_2_O_2_ and depolarization of the mitochondrial membrane in *Leishmania braziliensis* [13], and inhibition of the growth in *Babesia* spp. [9]. Previous reports also suggested that EGCg can non-selectively inhibit proteins [14]. EGCg can successfully induce growth arrest and apoptosis through numerous signaling pathways [15]. EGCg is a successful anti-carcinogenic drug because it has the capacity to regulate anti-apoptotic and apoptotic proteins, growth factors, protein kinases, cell cycle proteins and transcription factors [16]. Additionally, EGCg can cause mitochondrial collapse [17]. Furthermore, EGCg exhibited an antifolate property against *Stenotrophomonas maltophilia* [18], by inhibiting dihydrofolate reductase (DHFR), the enzyme that catalyzes the reduction of 7,8-dihydrofolate to 5,6,7,8-tetrahydrofolate, which plays a role in nuclear biosynthesis. Thus, inhibition of DHFR can disrupt DNA synthesis. *A. castellanii* has DHFR and the antifolate trimethoprim drug had shown anti-acanthamoebic activity [19]. Therefore, the trophicidal effect of EGCg may be attributed to its antifolate activity.

Although EGCg seems to have no significant inhibitory effect on *A. castellanii* encystation, it is still interesting to identify the ideal EGCg concentration that prevents *A. castellanii* trophozoites from encysting. If *A. castellanii* remains in the active trophozoite stage, it is more susceptible to treatment. However, only a 27.7% reduction in encystation was achieved by 1000 µM (*p* = 0.15), with 500 µM even promoting encystation by 36.3% (*p* = 0.06) compared to the negative control. These findings suggest that EGCg has limited potential benefits as an anti-encystation treatment, though the effect observed in both concentrations were not considered statistically significant. It is also important to find the reasons for the lack of anti-encystation effect by EGCg. The positive control used in the anti-encystation experiment, PMSF, binds to serine proteases, and so counteracts the proteolytic activity associated with the early stages of encystment [20]. In regard to EGCg, the proteolytic enzyme urokinase (uPA) can be inhibited by EGCg, which consequently blocks the His-57 and Ser-195 protease catalytic triad [21]. It is conceivable to presume that 1000 µM EGCg might have inhibited proteases secreted from *A. castellanii* trophozoites and therefore reduced the rate of encystation. Further investigations into the EGCg-mediated anti-encystation effect may improve our understanding of how EGCg works against *A. castellanii* encystment.

Cytotoxicity analysis showed that matcha had a high cytotoxic effect on cultured MDCK cells (Figure 3A), however EGCg had less cytotoxic impact, particularly at lower concentrations (Figure 3B). Maintaining mammalian cells in a health status in vitro requires the presence of growth factors, serum and other nutrients in the culture medium, which are essential for sustaining the physiology, metabolism and viability of cultured cells [22]. Culture medium used in the cytotoxicity testing of EGCg was prepared by dissolving the EGCg in the same cell culture medium DMEM used to maintain the MDCK cells. This has ensured that cultured MDCK cells have received all the nutritional needs that they normally obtain from DMEM to maintain their viability. By contrast, cytotoxicity testing of matcha involved the direct exposure of MDCK cells to matcha brew at a 100% concentration or diluted with DMEM medium to prepare lower concentrations for testing. Therefore, the increased cytotoxic effect of matcha brew on cultured MDCK cells might have been influenced or aggravated by the scarcity of nutrients in matcha brew used in the evaluation of matcha cytotoxicity.

The colorimetric SRB assay was deemed less reliable for EGCg testing. SRB absorbance relates to the quantity of proteins inside the cell, which indirectly correlates with the number of *A. castellanii* trophozoites present. Wells treated with higher EGCg concentrations were expected to yield lower absorbance values, but the reverse occurred. This gave a false impression that EGCg may have promoted the parasite’s growth. These observations might be attributed to an unintentional sedimentation of EGCg, because it can react with Ag^+^ and forms a brown aggregated nanoparticle sediment [23]. The PYG medium already contained traces of minerals and heavy metals, however, whether these minerals and metals contributed to the formation of EGCg precipitates remains to be investigated. The potential reactions between detection reagents and the tested chemicals may result in false positive and negative absorbance values [24]. Therefore, compounds used in a colorimetric assay must be checked for unexpected interactions with the dye used in the assay, in order to enhance the accuracy of the results [25].

FTIR fingerprint analysis showed that exposure of trophozoites to the matcha (hot or cold brew) induced changes in the glycogen and carbohydrates within trophozoites when compared to untreated trophozoites (control). A small difference in the carbohydrate region was also observed between trophozoites treated with cold and hot brews. Our results also showed a slight increase in glycogen content in the trophozoites treated with cold brewed, compared to those treated with hot brewed matcha. However, there were large variations between the samples. Exposure of *A. castellanii* to hot brewed matcha during encystation caused an increase in the carbohydrate, which is a main constituent of the cyst wall [26], suggesting that exposure to hot brewed matcha may have triggered the cyst wall formation. The rapid differentiation from trophozoites into pseudocysts enabled *A. castellanii* to survive lethal exposure to organic solvents [27] or contact lens solutions [28]. Therefore, the induction of encystation might be a mechanism employed by *A. castellanii* to offset the stress induced after treatment with a high concentration of matcha.

## 4. Materials and Methods

### 4.1. Chemicals and Reagents

The following reagents used in the present study were purchased from Sigma-Aldrich (Dorset, UK): sulforhodamine-B (SRB), yeast extract, proteose-peptone, (–)-epigallocatechin-3-O-gallate, chlorhexidine digluconate (CHX) solution, magnesium chloride, and acetic acid. Reagents purchased from Fisher Scientific UK Ltd. (Loughborough, UK) included: 10% D-(+)-glucose monohydrate, tris(hydroxymethyl)aminomethane, phenylmethylsulfonyl Fluoride (PMSF), and sodium dodecyl sulfate (SDS). Trichloroacetic acid (50% *w*/*v*) was purchased from Merck KGaA (Darmstadt, Germany).

### 4.2. Culture of A. castellanii Trophozoites

A clinical strain of the virulent *A. castellanii* T4 genotype (American Type Culture Collection; ATCC 30011) was maintained on an axenic peptone-yeast extract-glucose (PYG) medium at 25 °C. PYG media was prepared by dissolving 10 g of 10% D-(+)-glucose monohydrate, 7.5 g of yeast extract and 7.5 g of proteose-peptone in 500 mL of distilled water.

### 4.3. Preparation of C. sinensis Brews, EGCg and Encystation Solutions

Cold and hot brewed matcha. A cold brewed matcha was prepared by combining 2 g of Japanese organic matcha green tea powder with 200 mL of ambient temperature distilled water (previously boiled to sterilize and allowed to cool). This solution is equivalent to a 100% concentration (0.01 g matcha powder/mL water). The solution was occasionally mixed and allowed to brew for 24 h at 4 °C. A hot brewed matcha was prepared by combining 2 g of pure grade matcha powder to 200 mL of previously boiled distilled water. The solution was occasionally mixed and allowed to brew for 1 h at room temperature. After brewing, the supernatants were sterilized by filtration through a Minisart™ 0.2 µM filter (Sartorius, Göttingen, Germany) and stored in a falcon tube sealed with parafilm at 4 °C. Matcha-containing PYG medium was prepared by dissolving 10 g glucose monohydrate, 7.5 g of yeast extract and 7.5 g of proteose-peptone in 500 mL of already prepared sterile matcha brew. This was done to ensure that the base constituents of PYG medium are also available in the *C. sinensis*-PYG medium.

EGCg solution. A 1000 µM EGCg solution was prepared by dissolving 0.5 g EGCg in 220 mL of PYG medium and then filtered through Minisart™ 0.2 µM filters. Lower concentrations were prepared by using PYG medium.

Encystation solutions. Standard encystation solution was prepared by adding 10 g of D-(+)-glucose monohydrate and 0.48 g of magnesium chloride (MgCl) to 100 mL of distilled water. A 100 % matcha-based encystation solution was prepared by adding 10 g of glucose and 0.48 g MgCl to 100 mL of a previously prepared hot or cold brewed matcha, which was used as the solvent, instead of distilled water. The lower (50%) concentration was prepared by using standard encystation solution as the diluent. The 1000 µM of EGCg-containing encystation solution was prepared using standard encystation solution as the solvent. The lower (500 µM) concentration was prepared using the standard encystation solution as the diluent. All solutions were stored at 4 °C.

### 4.4. Evaluation of the Trophozoite Growth

The number of *A. castellanii* trophozoites was adjusted to 2.5 × 10^4^ trophozoites/mL. Then, 100 µL of the trophozoite suspension was seeded into each well of 96-well microtiter tissue culture plates (Thermo Fisher Scientific, Waltham, MA, USA). Then, brewed matcha (hot or cold) was added at serial concentrations (25%, 50%, 75% and 100%). After incubation for 3 days, the plates were stained using the SRB-based colorimetric assay as described previously [29]. Level of growth inhibition of *A. castellanii* trophozoites was evaluated by measuring the OD_492_ using a LT-4000 microplate reader (Labtech International Ltd., East Sussex, UK). To evaluate the effect of EGCg against *A. castellanii* trophozoites, two assays were performed: colorimetric SRB staining assay [29] and absolute manual counts. We tested the following concentrations of the EGCg: 25, 50, 75, 100, 250 and 500 µM. For the controls, cultures without EGCg (negative) and another containing CHX (positive) were used. Blank wells containing only 200 µL of PYG were used to calibrate the total absorbance. This experiment was repeated for a total of three experiments.

### 4.5. Inhibitory Effect on A. castellanii Cysts

A standard concentration of 5 × 10^5^ *A. castellanii* trophozoites was used to seed small T25 (25 cm^2^) NUNC™ tissue culture flasks (Fisher Scientific, Leicestershire, UK), maintained in 5 mL encystation medium. Matcha-containing encystation solution or EGCg-containing encystation solution were added to make up 50% and 100% (for matcha) and 1000 µM and 500 µM (for EGCg), respectively. We tested only hot brewed *C. sinensis* in this experiment, because in the previous trophozoite assay we found a negligible difference in the efficacy of cold and hot brewed matcha. Each flask was seeded with respective concentrations of encystation solutions, up to a total volume of 10 mL. For the positive controls, 5% (0.5 mL) of PMSF was added to a culture flask containing 9.5 mL of standard encystation solution. The negative control flask contained 10 mL of standard encystation solution only. After incubation for 3 days at 25 °C, the number of *A. castellanii* per ml in each flask were counted using a hemocytometer. After counting, 10 mL of 1% SDS was added to each flask (forming a 0.5% concentration), and the flasks were incubated at ambient temperature for 1 h. This ensures that any *A. castellanii* which are not fully encysted become digested, leaving only mature cysts in each flask [30]. After incubation, the number of cysts per 20 mL in each flask was counted using a hemocytometer. Encystation % was calculated using the formula (post-digestion number of cysts/pre-digestion number of cysts × 100). This experiment was repeated, for a total of three experiments.

### 4.6. Cytotoxicity Assessment

Madin Darby canine kidney (MDCK) cells were purchased from European Collection of Authenticated Cell Cultures (ECACC, Salisbury, UK). MDCK cells were maintained in Dulbecco’s modified Eagle’s medium (DMEM), supplemented with 10% fetal bovine serum and 1% PenStrep solution (100 U/mL penicillin and 100 μg/mL streptomycin). Cell cultures were incubated at 37 °C in a 5% CO_2_ atmosphere and passaged every 3 days. MDCK cells were seeded into the wells of 96-well tissue culture plates (Thermo Fisher Scientific, Waltham, MA, USA) at 5 × 10^3^/well. After 24 h of incubation, various concentrations of matcha (25%, 50%, 75% and 100%) and EGCg (25, 50, 75, 100, 250 and 500 µM) were prepared in DMEM and inoculated into wells. After incubation for 24, 48, or 72 h, the toxic effects of matcha and EGCg on MDCK cells were evaluated by measurement of the MDCK cell uptake of the SRB stain, as described previously [31]. MDCK cells with medium and without any treatment served as a control. Positive controls were generated by adding 3% SDS ~45 min before SRB staining. The absorbance was measured at 492 nm using a LT-4000 microplate reader. This experiment was performed in duplicate, and each concentration was tested using at least 4 technical replicates.

### 4.7. FTIR Microspectroscopy, Spectra Processing and Multivariate Data Analysis

We used FTIR microspectroscopy to analyze the chemical changes that occurred in the trophozoites and cysts in response to treatment with matcha.

#### 4.7.1. Preparation of the Samples

To characterize the effect of match on trophozoites treated with hot or cold brew matcha, ~5 × 10^5^ trophozoites were seeded in T-25 cm^2^ tissue culture flasks and were treated with 100% hot or cold brewed matcha. The negative control included trophozoites treated with PYG medium only. After 48 h, the trophozoite suspension was harvested and fixed in 4% paraformaldehyde (PFA), then washed twice and stored in isotonic saline solution at 4 °C until use. To study the effect of brewed matcha on the cyst formation, we seeded 5 × 10^5^ trophozoites in T25 (25 cm^2^) NUNC™ tissue culture flasks and were treated with 100% matcha-containing encystation solution. The negative control included trophozoites treated with a standard encystation solution alone. After 3 days, the cysts were harvested and washed in distilled water and fixed in 4% PFA. Then, fixed cysts were washed twice and stored in isotonic saline solution at 4 °C until use. Before the spectral measurement, samples (trophozoites and cysts) were mounted on a 1 mm thick CaF_2_ window by drop casting, followed by gentle rinsing in bi-distilled water briefly to remove any remaining salt crystals, and then thoroughly air-dried for ~30 min. *Acanthamoeba* (trophozoites and cysts) were found to be dispersed individually across the substrate when prepared in this way.

#### 4.7.2. FTIR Microspectroscopy

FTIR spectra of single trophozoites/cysts were collected using an infrared spectroscopic microscope (Spotloight^TM^ 400 FT-IR, PerkinElmer, Inc., Waltham, MA, USA) with a mercury cadmium telluride (MCT) detector. The samples were visually inspected under the microscope to locate the trophozoites/cysts for measurement. We randomly selected 44 to 70 undamaged trophozoites/cysts for analysis. Measurements of single trophozoites/cysts were collected in transmission mode with a 20 µm × 20 µm aperture at 8 cm^−1^ spectral resolution, 4000–750 cm^−1^ spectral range and 32 scans averaging. A clear region of the CaF_2_ substrate was used as the background with 128 scans averaging. The analysis was focused on the first three principal components, which contained over 85% of the variance in the data.

#### 4.7.3. Data Processing and Analysis

After collecting the spectra, spectral pre-processing was performed using the OPUS 7.8 software (Bruker Optics Ltd., Ettlingen, Germany). The spectra were first processed using the “scattering correction” with 64 baseline points, followed by truncating to 3800–850 cm^−1^ and then further baseline corrected using the “concave rubber band” method with 10 iterations and 64 baseline points. After baseline correction, spectra were vector normalized. A quality control step was also incorporated so that spectra with absorbance greater than 0.1 in the bio-silent region (2500–1800 cm^−1^) were rejected (fewer than 4 spectra were rejected in each case). The pre-processed data were then further studied using principal component analysis (PCA) using the PyChem software Version 3.0.5g Beta [32]. The principal component (PC) scores for each treatment group were averaged with the standard deviations and T-test calculated using Microsoft Excel^TM^ software.

### 4.8. Data Analysis

Statistical analysis was performed using GraphPad Prism 8.3.0 software (GraphPad Software, San Diego, CA, USA). One-way ANOVA test was performed in order to determine if there were any significant differences between the results. A post hoc Dunnett’s test was also performed to determine if there was a significant difference for each variable group when compared with the control group. For the cytotoxicity experiment, a two-way mixed ANOVA was first performed, followed by a one-way ANOVA and post hoc Tukey’s test at each time, to compare different variable groups and the control group. Data represent the mean ± the SD of three separate experiments. The asterisk (*) or no significance (ns) above each bar denotes whether there was a significance observed based on the results obtained from the relevant post hoc test. Statistical differences between groups were considered significant if the *p* value was ≤0.05, and this is indicated in the figures by asterisks (* *p* < 0.05; ** *p* < 0.01; *** *p* < 0.001).

## 5. Conclusions

Taken together, the study results showed that matcha and EGCg can inhibit the growth of *A. castellanii* trophozoites in vitro. However, the findings showed that only matcha had a substantial anti-encystation effect. FTIR analysis revealed significant changes in the chemical composition trophozoites and cysts after matcha treatment. Further studies are necessary to elucidate the exact mode of action of matcha and EGCg on *A. castellanii* and to examine how they may interact with anti-acanthamoebic drugs. Given the in vitro cytotoxic effect, further studies are also required to elucidate the bioavailability and safety profile of matcha and EGCg.
